# Properties of Boolean dynamics by node classification using feedback loops in a network

**DOI:** 10.1186/s12918-016-0322-z

**Published:** 2016-08-24

**Authors:** Yung-Keun Kwon

**Affiliations:** School of Electrical Engineering, University of Ulsan, 93 Daehak-ro, Nam-gu, Ulsan, 44610 Republic of Korea

**Keywords:** Boolean dynamics, Robustness, Perturbations, Feedback loop, Signaling networks

## Abstract

**Background:**

Biological networks keep their functions robust against perturbations. Many previous studies through simulations or experiments have shown that feedback loop (FBL) structures play an important role in controlling the network robustness without fully explaining how they do it. Hence, there is a pressing need to more rigorously analyze the influence of FBL structures on network robustness.

**Results:**

In this paper, I propose a novel node classification notion based on the FBL structures involved. More specifically, I classify a node as a *no*-*FBL*-*in*-*upstream* (*NFU*) or *no*-*FBL*-*in*-*downstream* (*NFD*) node if no feedback loop is involved with any upstream or downstream path of the node, respectively. Based on those definitions, I first prove that every NFU node is eventually frozen in Boolean dynamics. Thus, NFU nodes converge to a fixed value determined by the upstream source nodes. Second, I prove that a network is robust against an arbitrary state perturbation subject to a non-source NFD node. This implies that a network state eventually sustains the attractor despite a perturbation subject to a non-source NFD node. Inspired by this result, I further propose a perturbation-sustainable probability that indicates how likely a perturbation effect is to be sustained through propagations. I show that genes with a high perturbation-sustainable probability are likely to be essential, disease, and drug-target genes in large human signaling networks.

**Conclusion:**

Taken together, these results will promote understanding of the effects of FBL on network robustness in a more rigorous manner.

**Electronic supplementary material:**

The online version of this article (doi:10.1186/s12918-016-0322-z) contains supplementary material, which is available to authorized users.

## Background

It is well known that bio-molecular networks can keep their regulatory functions robust against various types of external or internal perturbations. For instance, the fate decision mechanism in a bacteriophage life cycle [1], the chemotaxis process in *Escherichia coli* [2], and segmental polarization in *Drosophila melanogaster* [3] were shown to be robust against noisy environments. It is more interesting that the dynamics of a biological network can be highly related to its structural characteristics [4]. In particular, many recent studies have shown that a feedback loop (FBL), a circular chain of interactions, can play an important role in controlling the robustness or susceptibility of networks [5, 6]. For instance, the negative FBL between MDM2 and p53 maintains an optimal level of p53 and creates appropriate dynamics of p53 expression level changes for a given level of DNA damage [7]. The Xenopus cell cycle is also robustly controlled against a certain level of perturbation with the help of several FBLs [8]. It was shown that a high proportion of coherently coupled FBLs can enhance the robustness of a network against state perturbations [9]. The number of FBLs involved at a node was also found to be positively correlated with the node’s functional importance [10]. Those simulation studies, however, cannot fully explain how the FBLs influence the network robustness. Hence, there is a pressing need to more rigorously analyze the relationship between FBL structures and network dynamics.

To measure network robustness, I herein use a synchronous Boolean network model [9, 11, 12] in which a node state is represented by a Boolean value, and the states of all nodes are synchronously updated at every discrete time step. Every network state moves to another state, and a series of consecutive transitions are represented by a network state trajectory that eventually converges to a fixed-point or cyclic attractor. The attractor can describe various dynamic behaviors in a biological system, such as multi-stability and oscillations. If a node state is perturbed, the trajectory might converge to a different attractor. Therefore, a network is considered robust if the attractor does not change against a perturbation. Some tools have been proposed to quantify the network robustness by simulating the state transitions after randomly initializing the node states [13–17]. They have a limitation in network size for analysis, though, due to the exponential complexity of attractor computation. Therefore, it is a critical issue to find analytic results that can identify trivial parts that do not require further computation of state transitions.

In this paper, I focus on the effects of an FBL on Boolean converging dynamics. A state of a node is propagated to other nodes along a path in a chain of consecutive interactions. Therefore, the state cannot be fed back to the original node if it is not involved in an FBL. In other words, the current state of a node will eventually disappear unless a downstream path constructs an FBL. From that idea, I developed an FBL-based notion to classify nodes in a network. In particular, I defined two sets of nodes, *no*-*FBL*-*in*-*upstream* (*NFU*) and *no*-*FBL*-*in*-*downstream* (*NFD*), according to whether the upstream and downstream paths, respectively, involve any FBLs and proved two theorems regarding NFU and NFD nodes. One is that every NFU node is always frozen irrespective of the initial states of other nodes. This implies that the converging values of all NFU nodes are eventually fixed to a value determined by the upstream source nodes. It also means that a network is likely to be susceptible to a perturbation subject to the source nodes. The other is that a network is robust against an arbitrary perturbation subject to a non-source NFD node. In other words, a network state eventually converges to the same attractor despite a state perturbation subject to a non-source NFD node. Inspired by those results, I further developed a perturbation-sustainable probability which indicates how likely it is that a perturbation effect will be sustained through a network state trajectory and showed that it can adequately identify functionally important genes, such as essential, disease-associated, and drug-target genes, in large human signaling networks. Taken together, all of these results will promote understanding of the effects of FBLs on Boolean converging dynamics and reduce the computational costs of state transition-based simulation tools.

## Methods

### Structural classification of nodes in a network

In this study, a biological network is represented by a directed graph *G*(*V*, *E*) where *V* = {*v*_1_, *v*_2_, ⋯, *v*_*N*_} is a set of nodes and *E* = {*e*_1_, *e*_2_, ⋯, *e*_*A*_} is a set of directed edges (interactions); an edge *e* ∈ *E* is an ordered pair of nodes (*v*_*i*_, *v*_*j*_) where *v*_*i*_, *v*_*j*_ ∈ *V*. I use some notions from graph theory, including FBL and upstream/downstream paths, to represent the biological networks as follows.

#### Definition

A node *u* is an input node of *v* if there exists an interaction from *u* to *v* (i.e., (*u*, *v*) ∈ *E*). In addition, in-degree of *v* means the number of input nodes of *v*.

#### Definition

A node *v* is a source node if in-degree of *v* is zero. It is assumed that the state of a source node is fixed to its initial value over all the time.

#### Definition

Given a network *G*(*V*, *E*), a *path P* of a length *L*(≥1) is represented by a sequence of ordered nodes *u*_1_*u*_2_ ⋯ *u*_*L* + 1_ with interactions from *u*_*i*_ to *u*_*i* + 1_ ((*u*_*i*_, *u*_*i* + 1_) ∈ *E* for∀ *i* ∈ {1, 2, ⋯, *L*}) with no repeated nodes except *u*_1_ and *u*_*L* + 1_. In addition, *P* is called a *feedback loop* (*FBL*) if *u*_1_ = *u*_*L* + 1_.

#### Definition

Given a network *G*(*V*, *E*), an *upstream* (resp., *downstream*) *path P* = *u*_1_*u*_2_ ⋯ *u*_*L* + 1_ of a node *v* ∈ *V* is a path in which the last (resp., *first*) node *u*_*L* + 1_ (resp., *u*_1_) is *v*. Note that if *P* is a feedback loop, then it is both an upstream and downstream path of *v*. In addition, *P* = *u*_1_*u*_2_ ⋯ *u*_*L* + 1_ is a *maximal upstream* (resp., *downstream*) *path* if there is no longer path such as *wu*_1_*u*_2_ ⋯ *u*_*L* + 1_ (resp., *u*_1_*u*_2_ ⋯ *u*_*L* + 1_*w*) for some *w* ∈ *V*.

Based on those terms, I define no-FBL-in-upstream and no-FBL-in-downstream nodes as follows:

#### Definition

Given a network *G*(*V*, *E*), a node *v* is called a *no*-*FBL*-*in*-*upstream* (*NFU*) (resp., *no*-*FBL*-*in*-*downstream*; *NFD*) node if there is no upstream (resp., *downstream*) path *P* = *u*_1_*u*_2_ ⋯ *u*_*L*_*v* (resp., *P* = *vu*_1_*u*_2_ ⋯ *u*_*L*_) such that for some *i* ∈ {1, 2, ⋯, *L*}, *u*_*i*_ is involved in any feedback loop.

**Fig. 1:**
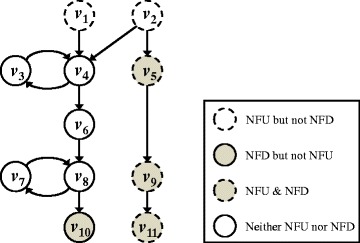
An example of NFU and NFD nodes in a network. Every node is classified into one of four categories: “NFU but not-NFD”, “NFD but not-NFU”, “NFU and NFD”, and “Neither NFU nor NFD”

Figure 1 shows an example of NFU and NFD nodes in a network. This network contains five NFU nodes *v*_1_, *v*_2_, *v*_5_, *v*_9_, and *v*_11_ and four NFD nodes *v*_5_, *v*_9_, *v*_10_ and *v*_11_. Note that *v*_5_, *v*_9_ and *v*_11_ are both NFU and NFD nodes. On the other hand, it also contains five nodes that are neither NFU nor NFD nodes because an FBL is both upstream and downstream of each of them. In particular, note that *v*_6_ is not directly involved in an FBL. In this paper, I will show that the NFU and NFD nodes can induce interesting dynamic properties in a perturbation analysis.

### A perturbation analysis in a Boolean network model

To define the robustness or sensitivity of a network, I use a synchronous Boolean network model used in previous studies [9, 12, 16]. In a Boolean network *G*(*V*, *E*), each *v*_*i*_ ∈ *V* has a value of 1 (on) or 0 (off) that represent the possible states of the corresponding elements. For example, the values 1 and 0 represent the “turn-on” and “turn-off” states of a gene, respectively. A directed interaction (*v*_*i*_, *v*_*j*_) can represent a positive (activating) or negative (inhibiting) relationship from *v*_*i*_ to *v*_*j*_. The value of each variable *v*_*i*_ at time *t* + 1 is determined by the values of *k*_*i*_ other variables ${v}_{i_1},{v}_{i_2},\cdots, {v}_{i_{k_i}}$ with an interaction to *v*_*i*_ at time *t* by a Boolean update function ${f}_i:{\left\{0,1\right\}}^{k_i}\to \left\{0,1\right\}$ where *f*_*i*_ is a constant value if *v*_*i*_ is a source node. All the Boolean variables are synchronously updated by a set of update functions *F* = {*f*_1_, *f*_2_, ⋯, *f*_*N*_}, and each update rule can be written as ${v}_i\left(t+1\right)={f}_i\left({v}_{i_1}(t),{v}_{i_2}(t),\cdots, {v}_{i_{k_i}}(t)\right)$.

Many studies have been performed to elucidate the dynamic behaviors of biological networks. In particular, I address robustness against perturbations in terms of Boolean dynamics. In a Boolean network *G*(*V*, *E*) , a *network state* at time *t* is represented by an ordered list **v**(*t*) = [*v*_1_(*t*), *v*_2_(*t*), ⋯, *v*_*N*_(*t*)] ∈ {0, 1}^*N*^. Then, for a subset *U* = {*u*_1_, *u*_2_, ⋯, *u*_*M*_} ⊆ *V*, a *subset state* of *U* at time *t* denoted by **v**_*U*_(*t*) = [*u*_1_(*t*), *u*_2_(*t*), ⋯, *u*_*M*_(*t*)] ∈ {0, 1}^*M*^ is an ordered list consisting of values *u*_1_ through *u*_*M*_ at time *t*. A state trajectory of *V* starts from an initial state and eventually converges to either a fixed-point or limit-cycle attractor. These attractors can represent diverse biological network behaviors, such as multi-stability and oscillation [18–20]. This notion of an attractor introduces robustness in terms of the Boolean network dynamics as follows. If a network sustains an attractor against a perturbation, it is called robust against the perturbation. This concept has been widely used [5, 21–24]. Here, I consider an initial-state perturbation. Given an initial state **v**(0) = [*v*_1_(0), *v*_2_(0), ⋯, *v*_*N*_(0)] at *t* = 0, an initial-state perturbation subject to node *v*_*x*_ ∈ *V* represents a situation in which **v**(0) is mutated to **v**^'^(0) = [*v*_1_(0), ⋯, 1 − *v*_*x*_(0), ⋯, *v*_*N*_(0)], *i.e*., the corresponding initial value is switched to $\overline{v_x}(0)$ (the negation of *v*_*x*_(0)). An initial-state perturbation represents the abnormal (or malfunctioning) status of a protein or gene caused by a mutation. The attractors to which **v**(0) and **v***'* (0) will converge can be compared to each other. The network is called robust or sensitive against the perturbation according to whether the attractors are the same as or different from each other, respectively. Based on this concept, I define some terms more rigorously with respect to the Boolean dynamics, as follows.

#### Definition

The sequence of states to which **v**(0) eventually converges is called the *attractor* induced from **v**, which is denoted by an ordered list of network states ζ(**v**) = [**v**(*τ*), **v**(*τ* + 1), …, **v**(*τ* + *p* − 1)] where **v**(*t*) = **v**(*t* + *p*) *for* ∀ *t* ≥ *τ* (*p* is a length of the attractor), and **v**(*i*) ≠ **v**(*j*) *for* ∀ *i* ≠ *j* ∈ {*τ*, *τ* + 1, …, *τ* + *p* − 1}. In addition, for a subset *U* ⊆ *V*, ζ(**v**_*U*_) = [**v**_*U*_(*τ*), **v**_*U*_(*τ* + 1), …, **v**_*U*_(*τ* + *p* − 1)] represents the states sequence of *U* in the attractor induced from **v**.

#### Definition

For *U* ⊆ *V*, *ζ*(**v**_*U*_) is *frozen* if there exists a time step *τ* such that *v*(*t*) = *v*(*t* + 1) for ∀ *v* ∈ *U and* ∀ *t* ≥.

#### Definition

Given two attractors with a same length, *ζ*(**v**) = [**v**(*τ*), **v**(*τ* + 1), …, **v**(*τ* + *p* − 1)] and *ζ*(**v**^'^) = [**v**^'^(*τ*^'^), **v**^'^(*τ*^'^ + 1), …, **v**^'^(*τ*^'^ + *p* − 1)], they are *equivalent* to each other if there exists a time step offset *t* ≥ 0 such that **v**(*τ* + *i*) = **v**^'^(*τ*^'^ + (*i* + *t*) *mod p*) for ∀ *i* ∈ {0, 1, …, *p* − 1}.

#### Definition

Consider an arbitrary initial state **v**(0) = [*v*_1_(0), *v*_2_(0), ⋯, *v*_*N*_(0)] and its perturbed state at *v*_*x*_ ∈ *V*, **v**^'^(0) = [*v*_1_(0), ⋯, 1 − *v*_*x*_(0), ⋯, *v*_*N*_(0)]. The Boolean network is called *robust* against the perturbation subject to *v*_*x*_ if *ζ*(**v**) is equivalent to *ζ*(**v**^'^). Otherwise, it is called *sensitive* or *susceptible*.

### Datasets of signaling networks and functionally important genes

In this study, I derive an estimated probability with which a perturbation effect is sustained in a network. To show the usefulness of it, I used two large-scale human signaling networks. One is the signaling network of 1659 genes and 7964 interactions constructed in a previous study [25] by integrating all the human signaling pathways in the KEGG (Kyoto Encyclopedia of Genes and Genomes) database [26] (see Additional file 1: Table S1). The other signaling network consists of 6306 genes and 62,937 interactions (version 6) downloaded from http://www.bri.nrc.ca/wang [27] (see Additional file 1: Table S2). In this work, I call them the KEGG and WANG networks, respectively. In addition, I considered essential, disease, and drug-targeted genes to represent functionally important genes. By using the DEG (Database of Essential Genes, version 5.4) database [28], I found 473 and 1519 essential genes included in the KEGG and WANG networks, respectively. I also found 403 and 1557 disease genes in the KEGG and WANG networks, respectively, using the OMIM (Online Mendelian Inheritance in Man) database [29]. Finally, I identified 353 and 1116 drug target genes in the KEGG and WANG networks, respectively, using the DrugBank database [30].

## Results

### Dynamic properties of NFU nodes

In this section, I show that NFU nodes are eventually frozen in Boolean network dynamics. A Boolean network *G*(*V*, *E*) with a set of update functions *F* is given.

#### Lemma 1

Given an initial state **v**(0) and a node *v*_*k*_ ∈ *V*, let *U* = {*u*_1_, *u*_2_, ⋯, *u*_*M*_} be the set of input nodes of *v*_*k*_. If *ζ*(**v**_*U*_) is frozen, then $\zeta \left({\mathbf{v}}_{\left\{{v}_k\right\}}\right)$ is also frozen.

#### *Proof*

For ∀ *u*_*i*_ ∈ *U*, there exists a time step *τ*_*i*_ such that *u*_*i*_(*t*) = *u*_*i*_(*t* + 1) for ∀ *t* ≥ *τ*_*i*_ since $\zeta \left({\mathbf{v}}_{\left\{{u}_i\right\}}\right)$ is frozen. Let $\tau =\underset{i\in \left\{1,2,\dots, M\right\}}{ \max}\left({\tau}_i\right)$. Then, *v*_*k*_(*t* + 2) = *f*_*k*_(*u*_1_(*t* + 1), ⋯, *u*_*M*_(*t* + 1)) = *f*_*k*_(*u*_1_(*t*), ⋯, *u*_*M*_(*t*)) = *v*_*k*_(*t* + 1) for ∀ *t* ≥ *τ*. Thus, $\zeta \left({\mathbf{v}}_{\left\{{v}_k\right\}}\right)$ is also frozen ■

#### Lemma 2

An initial state **v**(0) and a node *v* ∈ *V* are given. If every maximal upstream path of *v* includes at least one node *u* where *ζ*(**v**_{*u*}_) is frozen, then *ζ*(**v**_{*v*}_) is also frozen.

#### *Proof*

Let *P*_1_, *P*_2_, ⋯, *P*_*M*_ be the list of maximal upstream paths of *v*, and let *u*_*i*_ ∈ *P*_*i*_ (*i* = 1, 2, ⋯, *M*) be the node where $\zeta \left({\mathbf{v}}_{\left\{{u}_i\right\}}\right)$ is frozen. Then consider a sub-path *P*_*i*_^'^ of *P*_*i*_ starting from *u*_*i*_ and ending at *v*. Define *W* = {*w* ∈ *V*|*w* ∈ *P*_*i*_^'^ *for some i*} and let *l*(*w*) be the length of the longest path from *w* to *v*. Assuming that *L* = max_*w* ∈ *W*_*l*(*w*), *W* can be divided into *L* + 1 disjoint subsets *W*_0_, *W*_1_, ⋯ *and W*_*L*_ where *W*_*k*_ = {*w* ∈ *W*|*l*(*w*) = *k*}. Then *ζ*(**v**_{*w*}_) for ∀ *w* ∈ *W* is frozen by mathematical induction with respect to *l*(*w*), as follows. When *k* = *L*, it is obvious that *ζ*(**v**_{*w*}_) of every *w* ∈ *W*_*L*_ is frozen because *w* ∈ {*u*_1_, *u*_2_, …, *u*_*M*_}. Assume that *ζ*(**v**_{*w*}_) of every *w* ∈ *W*_*k* + 1_ is frozen and consider an arbitrary element *w*^'^ ∈ *W*_*k*_. Then every input node of *w*^'^ is an element of *W*_*k* + 1_. By lemma 1, $\zeta \left({\mathbf{v}}_{\left\{{w}^{\hbox{'}}\right\}}\right)$ is frozen. Thus, *ζ*(**v**_{*w*}_) of every *w* ∈ *W*_*k*_ is also frozen. By mathematical induction, *ζ*(**v**_{*w*}_) of every *w* ∈ *W* is frozen. Therefore, *ζ*(**v**_{*v*}_) is also frozen because *v* ∈ *W*■

Lemma 2 provides a sufficient condition for the frozenness of a node. This can be extended to the case of NFU nodes as follows.

#### Theorem 1

*An initial state***v**(0) *is given. If v* ∈ *V is an NFU node, then ζ*(**v**_{*v*}_) *is frozen*.

#### *Proof*

By the definition of NFU nodes, every maximal upstream of *v* starts with a source node *u* whose *ζ*(**v**_{*u*}_) is frozen. By Lemma 2, *ζ*(**v**_{*v*}_) is also frozen ■

This theorem implies that the states of NFU nodes are dependent on the states of source nodes, and this might make the network tend to be susceptible to perturbations subject to source nodes.

#### Corollary

An initial state **v**(0) is given. If there is no FBL then *ζ*(**v**) is frozen.

#### *Proof*

Since there are no FBLs, every *v* ∈ *V* is an NFU node. By Theorem 1, it follows that *ζ*(**v**) is always frozen irrespective of the initial states ■

Theorem 1 and its corollary explain the effect of FBLs on the frozenness of the converging state sequences. More specifically, every NFU node is frozen to a value determined by the set of source nodes included in its upstream paths. I also note that this result is strongly related to previous studies based on synchronous or asynchronous Boolean network models [31–33]. In particular, the corollary corresponds to a previous result having stated that the Boolean dynamics converges to a unique fixed point in an acyclic Boolean network [31]. It is also relevant to the previous results showed that limit-cycle attractors can be induced by negative feedback loops [32, 33]. In addition, Theorem 1 can reduce the computation of attractors in a large scale network by easily obtaining the converging values of the NFU nodes.

### Dynamic properties of NFD nodes

In the lemmas and a theorem of this section, I investigate the effect of FBLs on robustness. I consider an arbitrary initial state **v**(0) = [*v*_1_(0), *v*_2_(0), ⋯, *v*_*N*_(0)] and a perturbed state at *v*_*x*_ ∈ *V* from **v**(0), **v**^'^(0) = [*v*_1_(0), ⋯, *v*_*x* − 1_(0), 1 − *v*_*x*_(0), *v*_*x* + 1_(0), ⋯, *v*_*N*_(0)] in the following lemmas 3 and 4, and theorem 2. I denote the value of a node *w* ∈ *V* at time *t* in the trajectories starting from **v**(0) and **v**^'^(0) by **v**_{*w*}_(*t*) and **v**_{*w*}_^'^(*t*), respectively.

#### Lemma 3

Let **v**(0) be an initial state, **v**^'^(0) a perturbed state at *v*_*x*_ ∈ *V* from **v**(0), and *w* ∈ *V* an arbitrary node. If there is no path from *v*_*x*_ to *w* then **v**_{*w*}_(*t*) = **v**_{*w*}_^'^(*t*) for ∀ *t* ≥ 0.

#### *Proof*

The state value of node *w* is updated irrespective of that of node *v*_*x*_ because there is no path from *v*_*x*_ to *w*. Thus, the lemma holds ■

#### Lemma 4

Let **v**(0) be an initial state, **v**^'^(0) a perturbed state at *v*_*x*_ ∈ *V* from **v**(0), *w* ∈ *V* an arbitrary node. Let *Y* = {*y* ∈ *V*|*y is included in some path from v*_*x*_ *to w*} and *l*(*y*) the length of a longest path from *v*_*x*_ to *y* ∈ *Y*. If *v*_*x*_ is a non-source node and no node in *Y* is involved with any FBL, then **v**_{*w*}_(*t*) = **v**_{*w*}_^'^(*t*) for ∀ *t* ≥ *l*(*w*) + 1.

#### *Proof*

By mathematical induction with respect to *l*(*y*), I show that for every *y* ∈ *Y*, **v**_{*y*}_(*t*) = **v**_{*y*}_^'^(*t*) for ∀ *t* ≥ *l*(*y*) + 1, as follows. When *l*(*y*) = 0, it is obvious that *y* = *v*_*x*_. Then ${\mathbf{v}}_{\left\{{v}_x\right\}}(t)={{\mathbf{v}}_{\left\{{v}_x\right\}}}^{\hbox{'}}(t)$ for ∀ *t* ≥ 1 because *v*_*x*_ is a non-source node and involved with no FBL. To prove the inductive step, I assume that the property holds for *l*(*y*) ≤ *k* − 1. Consider an arbitrary *y* ∈ *Y* such that *l*(*y*) = *k* and let *U* be the set of input nodes of *y*. For every *u* ∈ *U*, there are two cases to consider: either *u* ∈ *Y* or *u* ∉ *Y*. In case of *u* ∈ *Y*, it is obvious that *l*(*u*) ≤ *k* − 1 by the definition of *l*(⋅). By the induction hypothesis, **v**_{*u*}_(*t*) = **v**_{*u*}_^'^(*t*) for ∀ *t* ≥ *l*(*u*) + 1. In case of *u* ∉ *Y*, it means that there is no path from *v*_*x*_ to *u*. Then **v**_{*u*}_(*t*) = **v**_{*u*}_^'^(*t*) for ∀ *t* ≥ 0 by lemma 3. From both cases, for ∀ *u* ∈ *U*, **v**_{*u*}_(*t*) = **v**_{*u*}_^'^(*t*) for ∀ *t* ≥ *k*. Then **v**_{*y*}_(*t*) = **v**_{*y*}_^'^(*t*) for ∀ *t* ≥ *k* + 1, thereby showing the property holds when *l*(*y*) = *k*. Since *w* ∈ *Y*, the lemma holds ■

#### Theorem 2

*Let***v**(0) *be an initial state*, **v**^'^(0) *a perturbed state at v*_*x*_ ∈ *V from***v**(0)*, and w* ∈ *V an arbitrary node. If v*_*x*_*is an NFD and non-source node, then the network is robust against a state perturbation subject to v*_*x*_.

#### *Proof*

I show that there exists a constant time *T* such that **v**_{*w*}_(*t*) = **v**_{*w*}_^'^(*t*) for ∀ *t* ≥ *T* in the following three cases. (*i*) Case that = *v*_*x*_ : Because *w* is an NFD and non-source node, **v**_{*w*}_(*t*) = **v**_{*w*}_^'^(*t*) for ∀ *t* ≥ 1. (*ii*) Case that *w* is not connected by any path from *v*_*x*_ : By lemma 3, **v**_{*w*}_(*t*) = **v**_{*w*}_^'^(*t*) for ∀ *t* ≥ 0. (*iii*) Case that *w* is connected by at least one path from *v*_*x*_ : Let *Y* = {*y* ∈ *V*|*y* is included in some path from *v*_*x*_ *to w*} and *l*(*w*) be a longest length of those paths, respectively. Because *v*_*x*_ is an NFD node, no node included in *Y* is involved with any FBL. By lemma 4, **v**_{*w*}_(*t*) = **v**_{*w*}_^'^(*t*) for ∀ *t* ≥ *l*(*w*) + 1. By (*i*),(*ii*), and (*iii*), there exists a constant time *T* such that **v**_{*w*}_(*t*) = **v**_{*w*}_^'^(*t*) for ∀ *t* ≥ *T* and ∀ *w* ∈ *V*. Accordingly, the attractors starting at **v**(0) and **v**^'^(0) are equivalent to each other. Therefore, the network is robust against the state perturbation subject to *v*_*x*_■

**Fig. 2:**
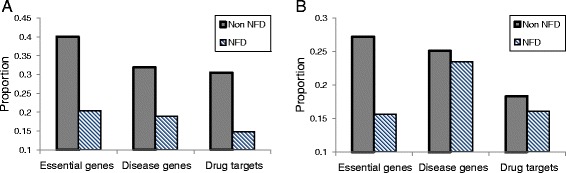
Comparison between groups of NFD and non-NFD genes in signaling networks. **a** Result in KEGG network with 975 NFD genes and 684 non-NFD genes. The proportions of essential genes in the NFD and the non-NFD groups were 0.204 and 0.401, respectively. The proportions of disease genes in the NFD and the non-NFD groups were 0.190 and 0.319, respectively. The proportions of drug-targets in the NFD and the non-NFD groups were 0.148 and 0.306, respectively. **b** Result in WANG network with 1706 NFD genes and 4600 non-NFD genes. The proportions of essential genes in the NFD and the non-NFD groups were 0.157 and 0.272, respectively. The proportions of disease genes in the NFD and the non-NFD groups were 0.235 and 0.251, respectively. The proportions of drug-targets in the NFD and the non-NFD groups were 0.161 and 0.183, respectively. In both networks, all the proportions for the NFD group were significantly smaller than those for the non-NFD group (all *p*-values<10^−10^)

Theorem 2 indicates that biological networks might be robust against perturbations subject to NFD nodes. To support this result, I compared NFD and non-NFD gene groups with respect to the proportions of essential genes, disease genes, and drug targets in two human signaling networks, the KEGG and WANG networks (Fig. 2; see Additional file 1: Tables S3 and S4 for details). As shown in Fig. 2, the proportions of essential genes, disease genes, and drug targets among NFD genes were significantly smaller than those among non-NFD genes in both networks (all *p*-values<10^−10^). I assume that essential genes, disease genes, and drug targets are likely to be susceptible to mutations, perturbations, or other external changes. In this regard, the relatively low proportions of essential genes, disease genes, and drug targets in the NFD group in the large-scale signaling networks support Theorem 2. In addition, I further examined the proportions of essential genes, disease genes, and drug targets in NFD group in random networks to examine if the observed result is specific to the signaling networks (see Additional file 2: Figure S1). I created each set of 100 random networks by rewiring the interactions of the KEGG (Additional file 2: Figure S1(A)) and WANG (Additional file 2: Figure S1(B)) networks so that the in-degree and the out-degree of the nodes are conserved, and observed that there is little difference between the NFD and non-NFD groups with respect to the proportions of essential genes, disease genes, and drug targets. This implies that the functionally important genes in the real signaling networks are not randomly distributed in terms of NFD classification.

### Estimation of sustainability of a perturbation effect

**Fig. 3:**
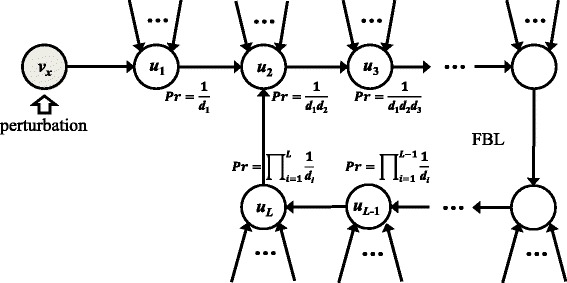
An example of a downstream path with a feedback loop. A node *v*_*x*_ is subject to a state perturbation, and the perturbation effect can be sustained at *u*_1_ with a probability of the inverse of the in-degree of *u*_1_. This propagation is continued along the path *P* = *v*_*x*_ *u*_1_ *u*_2_ ⋯ *u*_*L*_ involved with a FBL

In the previous section, Theorem 2 showed that a network state is robust against a perturbation as long as the perturbation effect is not sustained by downstream FBLs. In other words, the existence of downstream FBLs is a necessary condition to make a network susceptible to a perturbation. Inspired by that result, I have derived an estimated probability that a perturbation effect will be sustained. Given a node *v*_*x*_ ∈ *V* subject to a perturbation, Lemma 3 shows that only downstream paths of *v*_*x*_ need to be considered, and Lemma 4 shows that only those involved with an FBL need to be considered. I first estimate the probability with which a perturbation subject to *v*_*x*_ is sustained through a single path involved with a FBL. Figure 3 shows an example of a downstream path *P* = *v*_*x*_*u*_1_*u*_2_ ⋯ *u*_*L*_ of *v*_*x*_ which includes an FBL, and I consider **v**(0) and **v**^'^(0) which are an initial state and a perturbed state at *v*_*x*_ ∈ *V* from **v**(0), respectively. It is said that the effect of a perturbation starting at *v*_*x*_ at the initial time is sustained through propagations in a sequence of *u*_1_, *u*_2_, ⋯, *u*_*L*_ if ${\mathbf{v}}_{\left\{{u}_i\right\}}(i)\ne {{\mathbf{v}}_{\left\{{u}_i\right\}}}^{\hbox{'}}(i)$ for ∀ *i* ∈ {1, ⋯, *L*}. Herein, it is assumed that a probability with which *u*_*i*_(*i*) is differently updated by the flipped value of *u*_*i* − 1_(*i* − 1), denoted by $\Pr \left({\mathbf{v}}_{\left\{{u}_i\right\}}(i)\ne {{\mathbf{v}}_{\left\{{u}_i\right\}}}^{\hbox{'}}(i)\Big|{\mathbf{v}}_{\left\{{u}_{i-1}\right\}}\left(i-1\right)\ne {{\mathbf{v}}_{\left\{{u}_{i-1}\right\}}}^{\hbox{'}}\left(i-1\right)\right)$, is the inverse of the in-degree of *u*_*i*_ because of the following reason (for simplicity of explanation, *u*_0_ = *v*_*x*_ is assumed). Let $W=\left\{{w}_{1,}{w}_2,\cdots, {w}_{d_i}\right\}$ be the set of input nodes of *u*_*i*_ where *d*_*i*_ is the in-degree of *u*_*i*_. By assuming that the input nodes have an even degree of influence on updating *u*_*i*_, *i.e*., $\Pr \left(Y\Big|{X}_1\right)=\cdots = \Pr \left(Y\Big|{X}_{d_i}\right)$ where *Y* and *X*_*k*_(*k* ∈ {1, ⋯, *d*_*i*_}) denote two events ${\mathbf{v}}_{\left\{{u}_i\right\}}(i)\ne {{\mathbf{v}}_{\left\{{u}_i\right\}}}^{\hbox{'}}(i)$ and ${\mathbf{v}}_{\left\{{w}_k\right\}}\left(i-1\right)\ne {{\mathbf{v}}_{\left\{{w}_k\right\}}}^{\hbox{'}}\left(i-1\right)$, respectively. It is also assumed that *u*_*i*_ is always differently updated given a perturbation has occurred at one of the input nodes. Accordingly, $\Pr \left(Y\Big|{\mathsf{U}}_{k\in \left\{1,\cdots, {d}_i\right\}}{X}_k\right)= \Pr \left(Y\Big|{X}_1\right)+\cdots + \Pr \left(Y\Big|{X}_{d_i}\right)=1$, and thus Pr(*Y*|*X*_*k*_) = 1/*d*_*i*_ (∀ *k* ∈ {1, ⋯, *d*_*i*_}). Note that *u*_*i* − 1_ ∈ *W* because *u*_*i* − 1_ is one of the input nodes of *u*_*i*_. With this result, the probability with which the perturbation subject to *v*_*x*_ is sustained through a path *P* can be derived as follows:

1 $$ \Pr (P)=\left\{\begin{array}{cc}\hfill {\displaystyle {\prod}_{i=1}^L1/{d}_i,}\hfill & \hfill \mathrm{if}\ \mathrm{an}\ \mathrm{F}\mathrm{B}\mathrm{L}\ \mathrm{is}\ \mathrm{involved},\hfill \\ {}\hfill 0,\hfill & \hfill \mathrm{otherwise}\hfill \end{array}\right. $$

Let *P*_1_, *P*_2_, ⋯, *P*_*M*_ be the set of all downstream paths of *v*_*x*_. Then I define the perturbation-sustainable probability *γ*(*v*_*x*_), the probability that the perturbation subject to *v*_*x*_ will be sustained through at least one FBL, as follows:

2 $$ \gamma \left({v}_x\right)={ \max}_{i\in \left\{1,\cdots, M\right\}} \Pr \left({P}_i\right). $$

**Fig. 4:**
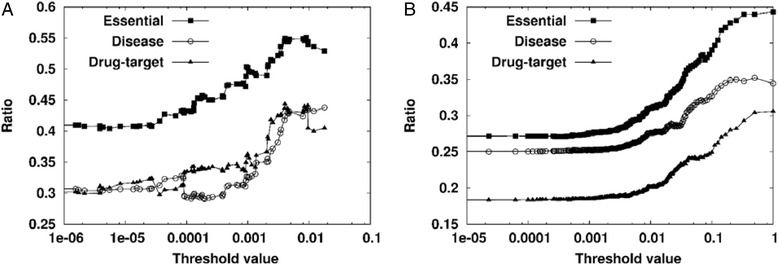
Changes in proportions of functionally important genes over the threshold value of the perturbation-sustainable probability. Given a threshold value *β*, the y-axis values indicate the proportions of essential genes, disease genes, and drug targets over the set of candidate genes whose perturbation-sustainable probability is larger than or equal to *β*. The larger the value of *β*, the smaller the number of candidate genes is. **a** Results of essential genes, disease genes, and drug targets in the KEGG network. For a reliable comparison, the maximal *β* was set to 0.0179 which generates 121 candidate genes. **b** Results of essential genes, disease genes, and drug targets in the WANG network. The maximal *β* was set to 1.0000, which results in 546 candidate genes

If a gene with a relatively high *γ*(*v*_*x*_) value is subject to a perturbation, the network is likely to induce an abnormal dynamics due to the well conserved perturbation effect. In this regard, the perturbation-sustainable probability can indicate how much a gene is functionally or dynamically important in a signaling network. To show the usefulness of this probability, I examined the relationship between *γ*(*v*_*x*_) and the proportions of putatively susceptible genes in human signaling networks (Fig. 4; see Additional file 1: Tables S3 and S4 for details). Given a threshold value *β*, the proportions of essential genes, disease genes, and drug targets among the set of genes such that {*v*_*x*_|*γ*(*v*_*x*_) ≥ *β*} are plotted against the threshold value in the KEGG (Fig. 4a) and WANG (Fig. 4b) networks. As shown in the figure, the genes with a high perturbation-sustainable probability are more likely to be essential genes, disease genes, and drug targets in both networks. This implies that the perturbation-sustainable probability can adequately identify the functionally important genes in human signaling networks. In addition, it is notable that the relation of the perturbation-sustainable probability to the functionally important genes was not observed in the random networks created by rewiring the interactions of the signaling networks (see Additional file 2: Figure S2). As in the results of Additional file 2: Figure S1, this also implies that the functionally important genes in the real signaling networks are not randomly distributed in terms of NFD classification. Taken together, it is interesting that such a simple topological measurement of genes based only on FBLs can efficiently predict the functionally important genes in human signaling networks.

## Discussion

In this study, I did not address the dynamics of non-NFU and non-NFD nodes, *i.e*., nodes that are involved in FBLs, and the analysis of their dynamics remains an open problem. In addition, the update-rule perturbation, another well-known type of perturbations, was not considered in this study because it influences the network robustness in a different way than the initial-state perturbation by changing the state transition diagram. Therefore, a future study should include analyses of genes that are neither NFU nor NFD nodes, and analysis of robustness against update-rule perturbations. Finally, it should be noted that the analyses in this study might not be effective for other types of biological networks than the signaling networks. For example, NFU/NFD classification was not meaningful in the large-scale gene regulatory networks [34, 35] because most genes were classified to NFD nodes. This implies that another method to further classify NFD nodes is required for analysis of those networks.

## Conclusions

It is well known that biological networks can keep their regulatory functions robust against external or internal perturbations. More interestingly, the network robustness is highly related to the network’s structural characteristics, including FBLs. However, previous results [2, 9, 10] have been presented mainly through simulation and experiment studies because of the complexity of real biological networks. That raised a pressing need to develop various analytical approaches to validate the promising conjectures. In this paper, I used a synchronous Boolean network model in which a node state is represented by a Boolean value and updated by a logical rule. A network is considered robust if the attractor does not change against a state perturbation. Based on that assumption, I created a novel concept to characterize the nodes with respect to FBL structures: *no*-*FBL*-*in*-*upstream* (*NFU*) and *no*-*FBL*-*in*-*downstream* (*NFD*). This FBL-based characterization is different from other FBL-based measures [10, 36] in that it focuses on involvement with FBLs in the upstream or downstream paths, not with the node itself. Based on that notion, I proved two simple but useful theorems. One is that an NFU node is always frozen irrespective of the initial states of other nodes. Thus, the converging dynamics of an NFU node can be simply determined. The other is that a network is robust against an arbitrary perturbation subject to a non-source NFD node. This result shows that a network state eventually converges to the same attractor despite a perturbation subject to non-source NFD nodes. Note that the two theorems hold for arbitrary update functions as well as initial states. In addition, the second theorem led me to develop a function to approximately compute the perturbation-sustainable probability. I verified its effectiveness by showing that the higher the probability, the larger the proportion of essential, disease-associated, and drug-target genes in human signaling networks. I believe these results will promote understanding of the effects of FBLs on network dynamics and reduce the cost of computing robustness in existing tools which simulate a network state trajectory [13–17].

## Additional files

Additional file 1: Table S1. Dataset of KEGG network. It consists of 1659 genes and 7964 interactions constructed by integrating all the human signaling pathways in the KEGG (Kyoto Encyclopedia of Genes and Genomes) database. **Table S2.** Dataset of WANG network. It consists of 6306 genes and 62,937 interactions downloaded from http://www.bri.nrc.ca/wang. **Table S3**. Detailed analysis result of the KEGG network. It includes information about essential genes, disease genes, drug targets, NRU/NRD genes, and perturbation-sustainable probabilities in the KEGG network. **Table S4.** Detailed analysis result of the WANG network. It includes information about essential genes, disease genes, drug targets, NRU/NRD genes, and perturbation-sustainable probabilities in the WANG network. (ZIP 352 kb) Additional file 2: Figure S1. Comparison between groups of NFD and non-NFD genes in random networks with respect to the proportions of essential genes, disease genes, and drug targets. In each subfigure, a set of 100 random networks were generated by rewiring the interactions of the signaling networks such that the in-degree and the out-degree of all nodes are conserved. (A) Result of random networks shuffled from KEGG network. The average numbers of NFD and non-NFD genes were 509 and 1150, respectively. The average proportions of essential genes in the NFD and the non-NFD groups were 0.2849 and 0.2852, respectively. The proportions of disease genes in the NFD and the non-NFD groups were 0.2417 and 0.2435, respectively. The proportions of drug-targets in the NFD and the non-NFD groups were 0.2141 and 0.2122, respectively. (B) Result of random networks shuffled from WANG network. The average numbers of NFD and non-NFD genes were 1544 and 4761, respectively. The proportions of essential genes in the NFD and the non-NFD groups were 0.2390 and 0.2413, respectively. The proportions of disease genes in the NFD and the non-NFD groups were 0.2455 and 0.2472, respectively. The proportions of drug-targets in the NFD and the non-NFD groups were 0.1768 and 0.1769, respectively. **Figure S2.** Changes in the proportion of functionally important genes over the threshold value of the perturbation-sustainable probability in random networks. In each subfigure, a set of 100 random networks were generated by rewiring the interactions of the signaling network such that the in-degree and the out-degree of all nodes are conserved. Given a threshold value *β*, the y-axis values indicate the average proportions of essential genes, disease genes, and drug targets over the set of candidate genes whose perturbation-sustainable probability is larger than or equal to *β* in random networks. (A) Results in random networks shuffled from KEGG network. For a reliable comparison, the maximal *β* was set to 0.0266 which generates 131 candidate genes on average. (B) Results in random networks shuffled from WANG network. The maximal *β* was set to 0.0908, which results in 121 candidate genes on average. (ZIP 362 kb)

## Abbreviations

FBL: Feedback loop
NFD: No-FBL-in-downstream
NFU: No-FBL-in-upstream
