# Supplementary material for: Properties of Boolean dynamics by node classification using feedback loops in a network
Source: BMC Syst Biol. 2016 Aug 24;10(1):83. doi: 10.1186/s12918-016-0322-z (PMC4997653; doi:10.1186/s12918-016-0322-z)
Supplement: Additional file 2: Figure S1. — Comparison between groups of NFD and non-NFD genes in random networks with respect to the proportions of essential genes, disease genes, and drug targets. In each subfigure, a set of 100 random networks were generated by rewiring the interactions of the signaling networks such that the in-degree and the out-degree of all nodes are conserved. (A) Result of random networks shuffled from KEGG network. The average numbers of NFD and non-NFD genes were 509 and 1150, respectively. The average proportions of essential genes in the NFD and the non-NFD groups were 0.2849 and 0.2852, respectively. The proportions of disease genes in the NFD and the non-NFD groups were 0.2417 and 0.2435, respectively. The proportions of drug-targets in the NFD and the non-NFD groups were 0.2141 and 0.2122, respectively. (B) Result of random networks shuffled from WANG network. The average numbers of NFD and non-NFD genes were 1544 and 4761, respectively. The proportions of essential genes in the NFD and the non-NFD groups were 0.2390 and 0.2413, respectively. The proportions of disease genes in the NFD and the non-NFD groups were 0.2455 and 0.2472, respectively. The proportions of drug-targets in the NFD and the non-NFD groups were 0.1768 and 0.1769, respectively. Figure S2. Changes in the proportion of functionally important genes over the threshold value of the perturbation-sustainable probability in random networks. In each subfigure, a set of 100 random networks were generated by rewiring the interactions of the signaling network such that the in-degree and the out-degree of all nodes are conserved. Given a threshold value β, the y-axis values indicate the average proportions of essential genes, disease genes, and drug targets over the set of candidate genes whose perturbation-sustainable probability is larger than or equal to β in random networks. (A) Results in random networks shuffled from KEGG network. For a reliable comparison, the maximal β was set to 0.0266 which generates 131 candi [file 12918_2016_322_MOESM2_ESM.zip › S1_Fig.pdf]

(A) (B)

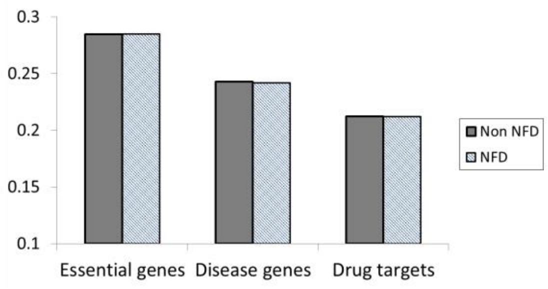

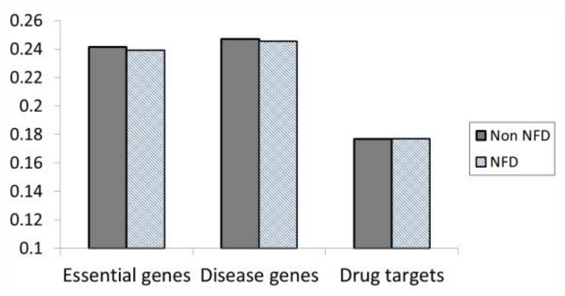

S1 Fig. Comparison between groups of NFD and non-NFD genes in random networks with respect to the proportions of essential genes, disease genes, and drug targets. In each subfigure, a set of 100 random networks were generated by rewiring the interactions of the signaling networks such that the in-degree and the out-degree of all nodes are conserved. **(A)** Result of random networks shuffled from KEGG network. The average numbers of NFD and non-NFD genes were 509 and 1,150, respectively. The average proportions of essential genes in the NFD and the non-NFD groups were 0.2849 and 0.2852, respectively. The proportions of disease genes in the NFD and the non-NFD groups were 0.2417 and 0.2435, respectively. The proportions of drug-targets in the NFD and the non-NFD groups were 0.2141 and 0.2122, respectively. **(B)** Result of random networks shuffled from WANG network. The average numbers of NFD and non-NFD genes were 1,544 and 4,761, respectively. The proportions of essential genes in the NFD and the non-NFD groups were 0.2390 and 0.2413, respectively. The proportions of disease genes in the NFD and the non-NFD groups were 0.2455 and 0.2472, respectively. The proportions of drug-targets in the NFD and the non-NFD groups were 0.1768 and 0.1769, respectively.
